# Evidence of epigenetic admixture in the Colombian population

**DOI:** 10.1093/hmg/ddw407

**Published:** 2017-01-10

**Authors:** Konrad Rawlik, Amy Rowlatt, María Carolina Sanabria-Salas, Gustavo Hernández-Suárez, Martha Lucía Serrano López, Jovanny Zabaleta, Albert Tenesa

**Affiliations:** 1 The Roslin Institute, Royal (Dick) School of Veterinary Studies, The University of Edinburgh, Easter Bush Campus, Midlothian, Scotland, UK; 2 MRC HGU at the MRC IGMM, University of Edinburgh, Western General Hospital, Crewe Road South, Edinburgh, UK; 3 Subdirección de Investigaciones, Instituto Nacional de Cancerología, Bogotá D.C., Colombia; 4 Departamento de Química, Universidad Nacional de Colombia, Bogotá D.C., Colombia; 5 Stanley S. Scott Cancer Center, Louisiana State University Health Sciences Center, New Orleans, LA, USA; 6 Department of Pediatrics, Louisiana State University Health Sciences Center, New Orleans, LA, USA

## Abstract

DNA methylation (DNAm) measured in lymphoblastoid cell lines has been repeatedly demonstrated to differ between various human populations. Due to the role that DNAm plays in controlling gene expression, these differences could significantly contribute to ethnic phenotypic differences. However, because previous studies have compared distinct ethnic groups where genetic and environmental context are confounded, their relative contribution to phenotypic differences between ethnicities remains unclear. Using DNAm assayed in whole blood and colorectal tissue of 132 admixed individuals from Colombia, we identified sites where differential DNAm levels were associated with the local ancestral genetic context. Our results are consistent with population specific DNAm being primarily driven by between population genetic differences in *cis*, with little environmental contribution, and with consistent effects across tissues. The findings offer new insights into a possible mechanism driving phenotypic differences among different ethnic groups, and could help explain ethnic differences in colorectal cancer incidence.

## Introduction

Phenotypic differences between human populations are widespread, however the relative contributions made by genetic and environmental differences between these populations remain largely unknown. In studies that compare populations of pure ancestry, the genetic and environmental population context are largely confounded, making it difficult to assess the role that either of these two components plays in shaping between population differences.

DNA methylation, which has been previously shown to vary across distinct populations (1–3), plays an important role in gene regulation (4), and hence has the potential to substantially contribute to the between population phenotypic variation. In general, variation of DNAm within a population has been demonstrated to have both significant genetic components (5,6) as well as strong associations with environmental factors (7,8). In admixed individuals, *cis* ancestral genetic context differs across DNAm sites. This naturally places DNAm sites of varying ancestral populations within the same environmental context, *i.e.* that of the admixed population. Admixed populations therefore offer a unique opportunity to disentangle the relative contributions of environmental and ancestral genetic factors.

Additionally, DNAm shows marked differences between tissues. Hence, although population specific DNAm (popDNAm) is well established in lymphoblastoid cell lines (LCL), it is unclear to what extent these population effects permeate different tissues. Consistent popDNAm differences across tissues would be a prerequisite for population specific DNAm contributing to ethnic differences in many clinically relevant phenotypes. We therefore examine popDNAm in two primary tissues, whole blood which represents an easily accessible tissue often employed in methylation studies and colon tissue which is of particular clinical relevance due to the potential role for methylation and population differences in colorectal cancer.

## Results

In order to identify ancestral popDNAm sites within an admixed population, we inferred local ancestry for all individuals and assigned locus-specific ancestral populations along the genome. This allowed us to compute a local ancestral context for individual DNAm sites in each individual and to test for associated differential methylation by regressing the methylation level at the site on this local ancestry. Specifically, the local ancestral context was defined as the proportion, across both haplotypes, of each of the ancestral populations based on inferred ancestries for markers located within a symmetric window centred at the site. By tagging ancestral haplotypes, marker specific ancestry can, in admixed populations, provide better tagging of between population genetic variations not assayed by SNP arrays. In an admixed population in contrast to regression on the actual genotype, which will capture the effects of variants common to all populations, regression on the local marker ancestry will in addition capture effects of population specific variants. As such local ancestry information has therefore been previously used to capture the contribution of population specific genetic variation in admixed individuals to complex phenotype heritability (9).

In this study, we considered a sample of 132 Colombian individuals from a larger case-control study (10) (Methods). Individuals were genotyped using the Illumina HumanOmniExpress Exome Chip and methylation was assayed in biopsies of healthy colorectal tissue in all individuals and additionally, in a subset of whole blood samples from 94 individuals using the Illumina Human Methylation450 BeadChip. We used RFMix (11) to estimate local ancestry for all individuals along the autosomal chromosomes using individuals from both the 1000 Genomes Project (12) and Human Genome Diversity Panel (HGDP) (13), to form African (YRI 1000 Genomes individuals), European (IBS 1000 Genomes individuals) and Native American (Colombian, Karitiana, Maya, Pima and Suri HGDP individuals, referred here collectively as NAM) reference populations (Methods). Averaging inferred local ancestries across all autosomal chromosomes we found estimates of ancestral proportions for each individual to be in line with previously reported ancestral proportions for Colombian individuals (14) (Supplementary Material, Fig. S1). In addition, we performed a PCA analysis of virtual ancestral genomes (15), *i.e.* for each individual we derive three ancestral genotypes by setting genotypes of all but one ancestral population as missing. The virtual ancestral populations segregated into three well separated clusters and co-clustered with the corresponding reference population (Supplementary Material, Fig. S2), further supporting the view that inferred local ancestries do indeed represent genomic segments originating from the three ancestral populations.

## Population-specific DNAm in normal colon tissue

**Figure 1.**
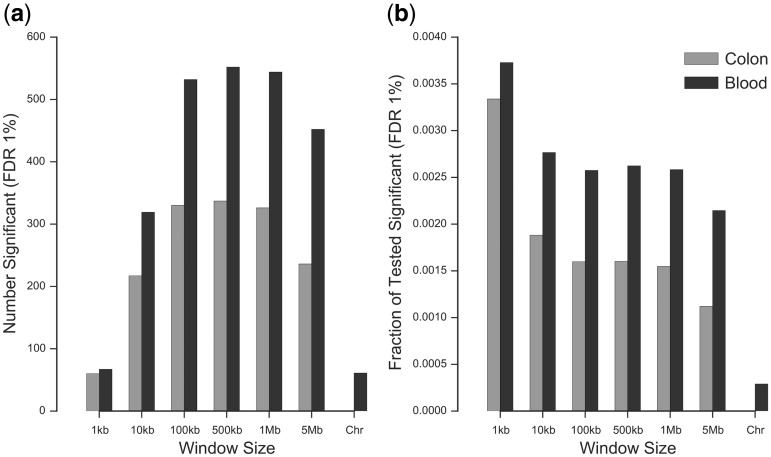
Summary of popDNAm sites across both tissues considered. We plot the numbers of sites with significant effects of local ancestry (FDR 1%) for different window sizes used for local ancestry estimation. Specifically, we show the absolute number of popDNAm sites (**A**) and the number of popDNAm sites as the fraction of sites tested for a specific window size (**B**).

**Figure 2.**
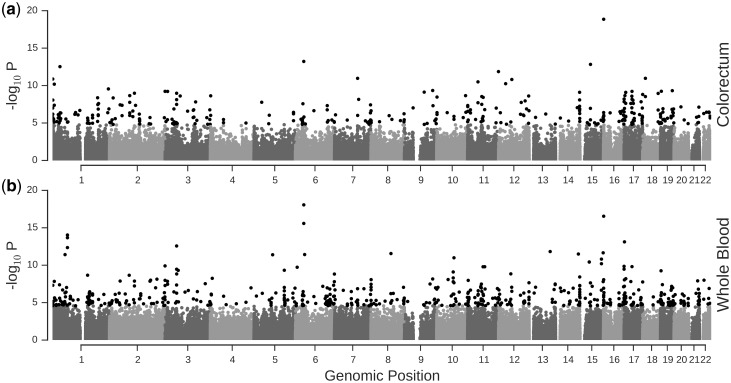
Distribution of identified popDNAm sites in the genome. We plot –log_10_ P value of the likelihood ratio test for presence of effects of local ancestry proportions in a 500kb for each tested DNAm site in colorectum (**A**) and whole blood (**B**). Sites which are significant (FDR 1%) are shown in black.

Estimates of heritability of DNAm indicate that a significant fraction of variation is due to *cis* effects. As local ancestry captures population specific variation, population specific DNAm driven by population specific genetic variation can be identified by associating local ancestry around the DNAm site with methylation levels. We first examined the effect the size of genomic window used for estimation of local ancestry has on identification popDNAm sites, which can be compared to the scales at which *cis*-heritability of DNAm sites has been identified in populations with a single ancestry. To this end, we used 210,724 autosomal CpG sites measured in colorectal tissue (Methods). Treating European ancestry as the reference, we regressed methylation levels, measured on the M-scale, on the African and Native American proportions of local ancestry, including age, gender, location of the biopsy, and recruitment city of the individual as covariates (Methods). We found that the number of sites showing a significant population effect, after a false discovery rate (FDR) adjustment (Methods), varied with the window size (Fig. 1A), with the highest number, 337 (FDR 1%), obtained for a 500kb window (Fig. 2A). However, taking into account the fact that the number of considered DNAm sites decreases with decreasing window sizes, due to the increasing presence of sites without any markers within smaller windows (Supplementary Material, Table S1), we found that the fraction of tested sites showing a population effect decreases steadily with window size (Fig. 1B). Importantly the sets of sites identified at different window sizes largely overlap (Supplementary Material, Fig. S3). In addition, comparing the distribution of numbers of markers per site within the 500kb windows, did not suggest that sites that showed an effect, *i.e.* popDNAm sites, had fewer markers than those that did not show an effect (Supplementary Material, Fig. S4). Including genome-wide proportions of ancestry as covariates did not change the results qualitatively (Supplementary Material, Fig. S5), although fewer sites with significant effects of local ancestry were detected, an expected effect due to possible confounding of local and genome-wide ancestry proportions. Results using a less stringent significance threshold, *e.g.* a FDR of 5%, which due to the low sample size may be preferable, yielded qualitatively similar results (Supplementary Material, Fig. S6). In contrast to estimates of *cis* heritability based on the genotype, which in admixed individuals captures the effect of variants common to all populations, local ancestry information captures population-specific genetic variation on the scale of the considered window. Therefore, our results suggest that popDNAm in colon is driven by population specific genetic variation in *cis*. Overall, these results are consistent with the observation of significant *cis* heritability for DNAm sites in populations of pure ancestry (5,6).

Examination of the location of identified popDNAm sites (Fig. 2) suggested the possibility of an enrichment of such sites at the telomeres. As local ancestry assignment is known to be less reliable at the ends of the chromosomes, this could potentially indicate a bias in our results. However while closer examination of the distribution of popDNAm sites relative to the telomeres indicated a slight increase in their frequency towards the ends of chromosomes, it did not differ significantly from other regions (Supplementary Material, Fig. S7).

## Genetic architecture at popDNAm sites

We examined the effect of local ancestry information in conjuncture with the observed genotype at sites identified as popDNAm sites in colon based on a 500kb window. To this end, we regressed DNAm levels at these sites on genotypes and local ancestry of SNPs within a 500kb window centred at the DNAm site. We find that all examined DNAm sites have at least one SNP with significant association (FDR 1%) of local ancestry with the methylation level. Similarly, a large majority of sites (314 of 337 sites) have at least one SNP at which the genotype is significantly associated (FDR 1%) with methylation levels. However, we find that for a majority (56%, 9633 of 17205) of DNAm-SNP pairs local ancestry provides a better model of DNAm variation, as assessed by model comparison based on the Akaike information criterion (16), than either the observed genotype, which was the best model for 24% of pairs, or a model including neither. Furthermore, for 25% SNPs (1799 of 7236 SNPs) for which we found a significant effect of local ancestry on a DNAm site, this effect remained significant (*P <* 0.05) when adjusting for the genotype at the SNP.

## Between tissue comparison of popDNAm

**Figure 3.**
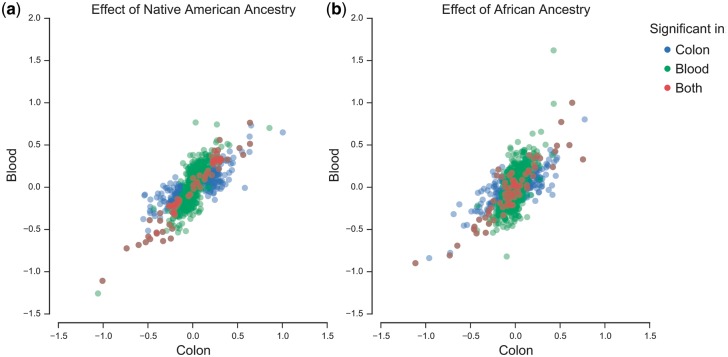
Across tissue comparison of ancestral population effects at individual popDNAm sites. We plot the estimated effect of local Native American (**A**) and African (**b**) ancestry within a 500kb window in whole blood tissue (Blood) versus the effect in colorectal tissue (Colon). Effects are only plotted for sites which, for either tissue, were identified as popDNAm sites based on local ancestry in the 500kb window. Colour indicates for which tissue a specific site was identified as exhibiting popDNAm.

Using methylation assayed in whole blood of 94 of the 132 Colombian individuals, we investigated whether the ancestral population specific methylation patterns identified in the colorectum are tissue specific. Following the same approach as for the colorectum (Methods) we obtained qualitatively similar results with respect to chosen window size (Fig. 1). However, the number of popDNAm sites was, despite the smaller sample size used in whole blood, generally higher in whole blood than in colorectum, *e.g.* for the 500kb window we identified 552 sites in whole blood and 337 in colorectum (FDR 1%). Only 69 sites were identified across both tissues (Fig. 2). However, comparing sites identified using a stringent significance threshold in both tissues, may underestimate the overlap as sites significant in one tissue may be only marginally non-significant in the other. We therefore follow a previously proposed approach (17) and evaluate the fraction of popDNAm sites identified in one tissue at a stringent threshold which are identified as popDNAm sites at a less stringent threshold in the other tissue. Considering popDNAm sites in colon identified using a 500kb window at FDR 1% we find that 75%, *i.e.* 253 DNAm sites, show a significant effect of local ancestry at a FDR of 50% in whole blood. Furthermore, comparing the estimated effects of both local Native American and African ancestry proportions, we found that while correlations across all sites were only modest (ρ = 0.22 for Native American and ρ = 0.15 for African effects), these where substantially higher when only considering sites which showed significant effects in at least one of the two tissues (ρ = 0.76 for Native American and ρ = 0.66 for African effects) or just the sites identified in both tissues (ρ = 0.97 for Native American and ρ = 0.90 for African effects) (Fig. 3). This suggests that ancestral population specific effects are to a large degree consistent across the two tissues.

## Effective sample size of admixed population

To estimate the sample size of a comparison across distinct populations corresponding to our admixed sample, we compute for each DNAm site the number of individuals of largely pure ancestry, *i.e.* individuals of more than three-quarter local ancestry for one population. We find that our study corresponds, on average, to an effective sample of 38, 18 and 5 individuals of European, Native American and African origin (Supplementary Material, Fig. S8) and is thus significantly smaller than previous studies. Furthermore, a re-analysis of the data of Moen *et al.* (1) (Methods) shows that the number of sites we identified is consistent with a sample size of 20 individuals per population (Supplementary Material, Fig. S9).

## Genomic context of population-specific DNAm

**Figure 4.**
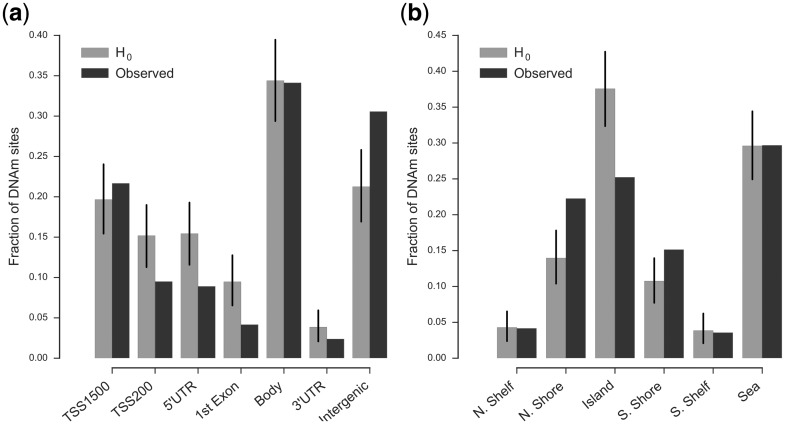
Enrichment of popDNAm sites in colorectum for genetic context. Specifically, gene context (**A**) and CpG-Island context (**B**). We plot the observed fraction of sites associated with a specific context amongst popDNAm sites and the expected such fraction under the assumption that popDNAm sites are randomly distributed amongst all tested sites ($H_{0}$). Error bars for $H_{0}$ indicate the empirically estimated 95% interval for this model. Considered popDNAm sites showed a significant effect based on 500kb window in colorectum.

The relation of DNAm sites to various other genomic features plays an important role in the potential function these sites can fulfill, with sites proximal to the promoter regions of genes being of particular interest due to the role they can play in regulating gene expression (4). We therefore examined whether popDNAm sites, identified in the colorectum using a 500kb window, are enriched in specific regions defined by their relation to genes and CpG-Islands (Methods). We found population specific sites to be depleted in regions at the 5’ end of genes, specifically, in the 200bp region upstream of the TSS (P = 0.0009), the 5’UTR (P = 0.0002) and the 1^st^ Exon (P = 0.0001), whilst being enriched in the intergenic context (*P <* 0.0001) (Fig. 4A and Supplementary Material, Table S2). We also found population specific sites to be depleted in CpG Islands (*P <* 0.0001), whilst being enriched on CpG-Island shores (*P <* 0.0001 and P = 0.0044 for N. and S. shores respectively), *i.e.* 2kb regions immediately up and down stream of CpG-Islands (Fig. 4B and Supplementary Material, Table S3). Results in blood were largely consistent with a strong enrichment for sites distal to CpG-Islands which had not been observed in the colorectum (Supplementary Materials, Fig. S10, Tables S2 and S3). Performing the same analysis using popDNAm sites identified using a less stringent threshold (FDR 5%) or excluding sites which are within 2Mb of the ends of chromosomes did not alter the results qualitatively (Supplementary Materials, Figs S11 and S12). These results contrast with previous reports of enrichment of popDNAm sites at gene associated regions (1), which however were based on comparisons of populations of pure ancestry and hence potentially suffered from confounding of environmental and genetic effects. Our results could therefore potentially indicate that popDNAm sites induced by environmental effects are enriched at DNAm sites in the proximity of genes, a hypothesis which testing will require further research. On the other hand, considering the previously discussed lower number of effective samples in our study, these results also raise the possibility that the power to detect population specific DNAm varies with genomic context. We also found popDNAm sites to be enriched (P = 0.02 and P = 0.01 for colon and whole blood respectively) for proximity to SNPs contained in the GWAS catalog (18), *i.e.* containing at least one such SNP in a 100kb window centred on the popDNAm site.

## Discussion

Previous studies identifying population specific DNAm have been performed using LCLs from individuals of pure ancestral backgrounds. Extrapolation of results from single transformed cell lines to primary tissue is complicated and our results may provide a better picture of popDNAm in primary tissues of clinical relevance. We therefore examined potential differences, and the sources of those differences, between our results and those reported previously in transformed cell lines. The number of identified sites showing a population effect in both colon and blood, as a fraction of all sites considered, is significantly lower than that identified in these studies. For example, 13% of tested sites have been reported to be differentially methylated between lymphoblastoid cell lines from individuals of European and African ancestry (1) compared to <1% in this study. Several potential explanations exist for the relative lack of identified sites in our study. On the one hand an excess of population specific sites could have been identified in previous comparisons of distinct populations due to the lack of control for different environmental backgrounds or technical differences in sample processing, which does not arise in our sample of admixed individuals. On the other hand, the discrepancy could be a consequence of differences in either the tissue or ancestral populations interrogated. However, the estimates of effective sample sizes suggest that a majority of the discrepancy in identified sites is attributable to the lower effective sample size of our study.

It is well documented that variation in proportion of cell types in heterogeneous tissues, like, *e.g.* whole blood, can affect analyses of methylation (19). We adjusted our analysis in whole blood using estimates of cell proportions based on reference data. As the reference panel may not have included individuals from our specific population or even from all of the ancestral populations contributing to our population, these estimates may be biased. While reference free methods have been proposed to adjust for variation in cell type proportions in epigenome wide association studies when association of one variable, *e.g.* a phenotype, with all DNAm sites is tested simultaneously (20), these methods are not directly applicable when testing for association with different variables for each DNAm site, as is the case here. However, variation in cell type proportion is unlikely to introduce a systematic bias in our results. For one, individuals in our study are not sampled from distinct populations, hence largely ruling out between population variations in cell proportions. Furthermore, any factor affecting the proportions of cell types will be constant for all DNAm sites within an individual. However local ancestry of DNAm sites varies across the genome, hence methylome, and is therefore randomized with respect to these factors across DNAm sites.

Our analysis of ancestral signatures of DNAm in an admixed population, demonstrates that popDNAm is driven by between population genetic variation in *cis* with the popDNAm sites, with largely concordant effects across the two tissues examined. The differences between ancestral populations are of particular interest in the context of previous results, in the same population, linking proportions of these ancestral populations to colon disease phenotypes (10). More generally, the approach followed in this study is not restricted to DNAm, but is more widely applicable to studying low level phenotypes, provided these are localised in the genome, allowing them to be associated with a local ancestral context, as is for example the case for gene expression. It will therefore provide a means to further dissect the relative environmental and genetic contributions to the basic mechanisms underlying ethnic phenotypic differences.

## Materials and methods

### Samples

Colorectal tissue and whole blood samples were obtained for 132 nominally unrelated Colombian individuals from a larger case-control study. Individuals were recruited in six cities in Colombia with the diagnosis of hyperplastic polyps, adenomas or adenocarcinoma of the colorectum. Details regarding recruitment and ascertainment have been previously presented (10). The study had ethical approval from the Ethics Board of The National Cancer Institute of Colombia, was conducted in accordance with the approved guidelines, and participants gave informed written consent.

### DNA methylation

DNA methylation was measured on samples of healthy colorectal tissue of all 132 individuals. Subsequently, methylation in whole blood was measured for 94 of these individuals, selected taking into account gender balance, genotyping and colorectal tissue methylation measure quality. Methylation levels were interrogated at 485,577 CpG sites across the genome using the Illumina HumanMethylation450 BeadChip. Raw methylation data processing and quality control were performed using the minfi R package (21) and data for both tissues was jointly normalised. To summarise the procedure, we excluded samples for which in excess of 5% of probes were not detected above background levels of variation (p = 0.01) or where the recorded sex did not match sex estimated based on methylation levels of probes on the X and Y chromosome. Individual probes were removed if they were not detected above background levels of variation in more than 5% of arrays or if they contained SNPs in the probe or had been found to be cross-reactive (22). We performed across array normalisation by stratified quantile normalization (23). In order to correct for possible technical variation between arrays we applied the ComBat algorithm (24).

The proportions of different cell types for whole blood samples were estimated using the method and reference data of Houseman *et al.* (19).

### Genotypes

The quality control, which followed a standard quality control procedure (25), has been described previously (6). In brief, we ascertained that for all samples no more than 5% of SNPs did not genotype. Based on the application of three successive filters, SNPs were removed if 1) they failed to type in more than 5% of samples or 2) if they were out of Hardy Weinberg Equilibrium (*P <* 0.0001) or 3) if they had a MAF less than 0.01. Additionally, the inbreeding coefficient for each sample was calculated from SNPs along the X chromosome and concordance of reported and inferred genetic sex ascertained and where discordant samples removed.

### Local ancestry inference

In order to infer local ancestries we constructed a reference panel for the three ancestral populations using individuals from the 1000Genomes Project (12) and Human Genome Diversity Panel (HGDP) (13). Specifically, we used the 107 Iberian individuals from Spain (IBS) as European reference and 108 individuals from Yoruba (YRI) as African reference. As Native American reference we used 108 individuals from five Central and South African populations (Colombian, Karitiana, Maya, Pima, Suri) from the HGDP.

We performed phasing of the 132 genotypes in our sample and those in the HGDP using shapeit (26) with the entire 1000Genomes Phase 3 panel (12) as reference. HapMap samples where obtained in phased form. After phasing we extracted the subset of 269,774 autosomal SNPs shared across all three sample sets, *i.e.* HapMap, HGDP and our Colombian genotypes.

Using the phased genotypes we inferred local ancestry using the RFMix algorithm (11). RFMix trains random decision forests using reference panel data to distinguish ancestry in small genomic windows. Predicted ancestries for individual windows are then combined with a hidden Markov model to produce local ancestries across entire chromosomes. An advantage of RFMix is its ability to infer local ancestries based on reference panels which exhibit some admixture themselves, a situation often encountered with Native American reference panels. To this end, RFMix utilises an EM approach, where after initial prediction for the admixed individuals, these individuals are combined with the reference samples, and then local ancestry is inferred for the combined sample. To correct for admixture in our reference, we performed five EM iterations, a number which has been found sufficient for optimal results (11). As expected, we found a non-negligible level of admixture amongst individuals in the Native American reference (Supplementary Material, Fig. S1).

### Identification of sites with population specific methylation

In order to identify sites exhibiting population specific methylation we proceeded as follows. For a methylation site at genomic position l we identified the set of SNPs within the region $\left[l-\frac{1}{2}\omega ,\;l+\frac{1}{2}\omega \right]$ where ω is a chosen window size. We computed $\pi_{i}$, the proportion of local ancestry for an ancestral population i, based on these SNPs. Specifically, we counted the number of SNPs, across both haplotypes of an individual, for which the inferred ancestry was i, and divided by twice the number of SNPs, *i.e.* the number of alleles. We then fitted a linear model regressing methylation on the M-scale on relevant covariates and the proportions of local Native American and African ancestry, so that the associated effects can be interpreted as the mean methylation difference between an individual of pure European and Native American or African ancestry respectively. We assessed the significance of the contribution of ancestral population to methylation by performing a likelihood ratio test with 2 degrees of freedom that compares the full model with a reduced model that includes only the covariates.

As covariates we included age and gender due to the well documented effects these have on methylation (7) and city of recruitment. The later was included as a proxy for potential within population differences in the environmental background between the samples. As colorectal biopsies had been taken from varying location and due to the documented differences in methylation between left and right colon (7), we included the origin of the tissue sample, left colon (includes descendant colon, sigmoid, sigmoid-rectum union and rectum), right colon (includes cecum, ascendant colon and transverse colon until splenic flexure) or nonspecific (NAs), as a further covariate in the analysis of the colorectal data. For the whole blood data meanwhile we included estimated proportions of cell types in the sample for CD4+ and CD8+ T-cells, natural killer cells, monocytes, granulocytes, and B-cells.

In all analyses, we applied a Benjamini and Hochberg false discovery rate adjustment at the level α=0.01.

### Genotype methylation associations

For each of the 337 popDNAm sites identified in colon using a window of 500kb at a FDR of 1%, we considered all SNPs for which we had inferred local ancestry information and which were within 500kb of the DNAm site. For each of the 11362 pairs of DNAm site and SNP, we fitted several linear models for methylation levels. All models included the same covariates as were used in identification of popDNAm sites. Specifically, we fitted a null model which did not contain further variables beyond the covariates, a genetic model which included the genotype coded as the number or reference alleles and a local ancestry model which contained the number of alleles of Native American and African ancestry as variables. We first identified pairs with either significant local ancestry or genetic effect by likelihood ratio tests between the two latter models and the null model, applying a Benjamini and Hochberg false discovery rate adjustment at the level α=0.01 for each of the analyses. We computed the Akaike information criterion (16) for each of the three models and used it to identify the best model for each DNAm-SNP pair. Finally we fitted, for each DNAm-SNP pair for which we had found a significant effect of ancestral population, a linear model containing both the coded genotype and the number of alleles of Native American and African ancestry. We then compared this model with the model containing just the genetic effect using a likelihood ratio test, in order to assess the significance of local ancestry when adjusting for the genetic effect.

### Effect of sample size on number of identified sites

We obtained the raw data of Moen *et al.* (1) for analysis of population specific methylation in HapMap CEU and YRI individuals (GEO series number GSE39672). This dataset consist of methylation measures in lymphoblastoid cell lines of 74 unrelated YRI and 60 unrelated CEU individuals, obtained with the Illumina HumanMethylation450 BeadChip. We followed the QC procedure described previously for the Colombian samples. Using this data, we simulated datasets of sizes 10, 20, 30, 40 or 50 individuals per population and computed the number of sites showing a population effect. For each sample size, we performed 10 repetitions with different random subsets. Sites with a population effect were identified by regressing methylation levels on the M-scale on the only available covariates which were gender and population identity.

### Enrichment of population-specific sites for genomic context

We used information contained in the manifest file of the Human Methylation450K array to assign methylation sites to a genomic context. This information identifies the relation of individual sites to genes. Specifically, a site is described as being in one of the following regions: either 200–1500 bp or 0–200 bp upstream of the TSS, in the 5’UTR or 3’UTR, in the first Exon or Body of the gene. The relation of a site to multiple genes can be included. In our analysis, we assign a site to a category if it is in the relation for at least one gene, with all sites which have no relations to any genes grouped in an Intergenic category. Note that sites can therefore be assigned to multiple categories, if they are in different relations with different genes. For evaluation of the relation to CpG-Islands we used regions as defined by Bibikova *et al.* (27), *i.e.* sites were assigned to be either in a CpG-Island, in the 2kb flanking regions (referred to as north and south shore for up- and downstream respectively), in regions 2–4 kb up- or downstream of an island (north and south shelf respectively) or the sea covering all remaining sites.

Given the assignment of sites to categories, enrichment was computed by comparing the fraction of sites showing a population effect within a category against the fraction of all sites tested within that category. Significance was assessed by comparing against the enrichment computed in 10000 draws from the null hypothesis. Specifically, we sampled 10000 random subsets of the entire set of tested sites, each matching the size of sites showing population effects.

Using SNPs contained in the GWAS catalog (18), we computed the number of popDNAm sites which had at least one proximal SNP, defined as a SNP within 50kb of the DNAm site. Enrichment for proximal SNPs was assessed as follows. We sampled 10000 random alternative DNAm site sets by sampling sets of size equal to the number of observed popDNAm sites from amongst all DNAm sites tested for population effects. Then, we computed an empirical P value for observing a set with more DNAm sites with a proximal SNP than observed for popDNAm sites.

All enrichment analyses were performed using sites which showed a significant (1% FDR) population effect for a 500kb window in either colorectal or whole blood tissue.

## Supplementary Material

Supplementary Material is available at *HMG* online.

## Supplementary Material

Supplementary Data
