# Supplementary material for: Evidence of epigenetic admixture in the Colombian population
Source: Hum Mol Genet. 2017 Jan 10;26(3):501–8. doi: 10.1093/hmg/ddw407 (PMC5409088; doi:10.1093/hmg/ddw407)
Supplement: Supplementary Data [file ddw407_Supp.docx]

**Evidence of epigenetic admixture in the Colombian population**

**Supplementary Material**

**Supplementary Figure 1.** **Proportions of ancestral populations in the study sample.** We plot each individual in the three reference populations (IBS, YRI and NAM) and the sample (COL) as vertical lines divided into the fractions of genome assigned by RFMix to three ancestral populations (**a**). For individuals in the study sample we also plot the distribution of the proportions of ancestry (**b**).


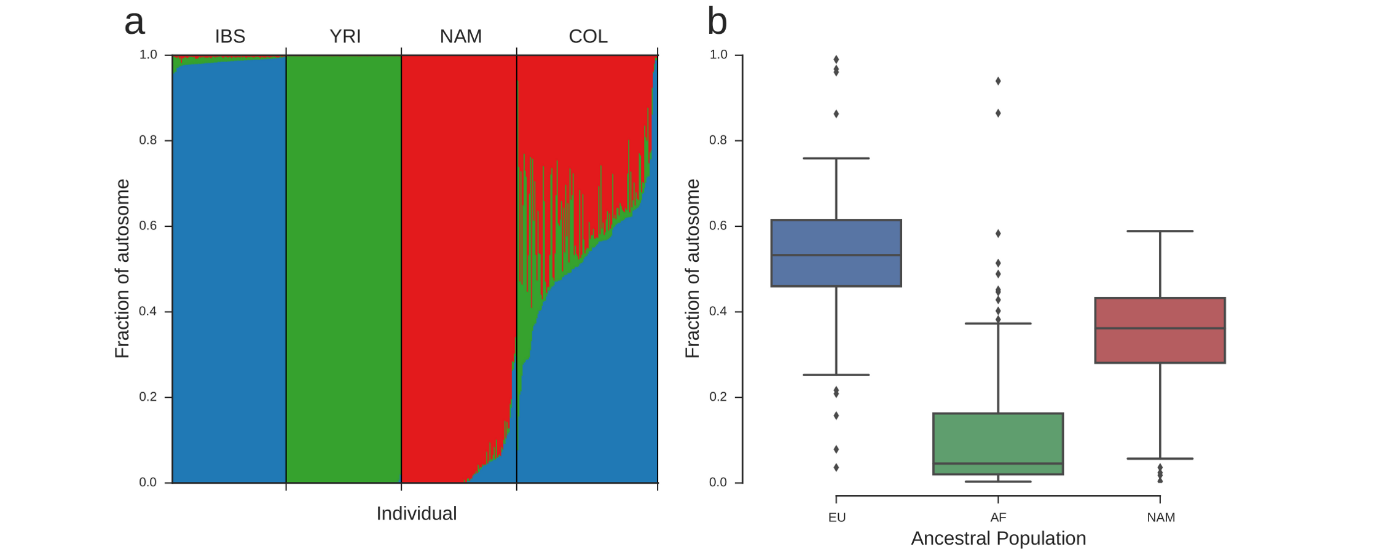


**Supplementary Figure 2.** **PCA analysis of virtual ancestral genomes of the study sample.** Virtual genomes consist of genotypes of admixed individuals from one ancestry component, i.e., with genotypes for markers inferred to originate from two of the ancestries set to missing. Only virtual genomes with missingness below 75% were included. Besides the virtual genomes, we included the entire admixed genomes of study individuals and genomes of individuals in the reference populations (not shown). Virtual genomes co-clustered with their respective reference population.

**
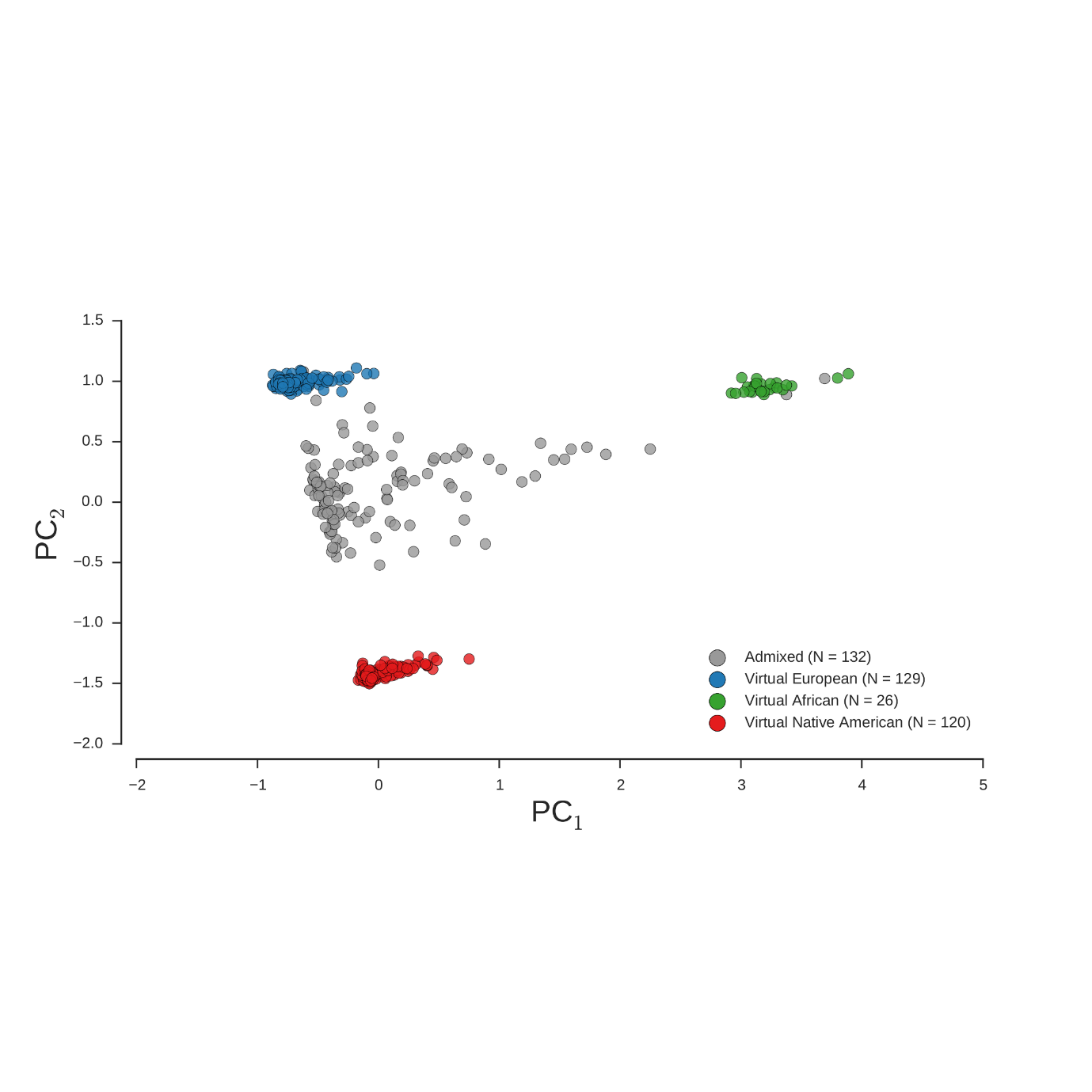
**

**Supplementary Table 1. Numbers of DNAm sites passing QC and containing at least one SNP for each window size and tissue.**

|  | **DNAm sites** |
| --- | --- |
| **1kb** | 17974 |
| **10kb** | 115348 |
| **100kb** | 206605 |
| **500kb** | 210416 |
| **1Mb** | 210622 |
| **5Mb** | 210716 |
| **Chr** | 210724 |

**Supplementary Figure 3. Distribution of identified popDNAm sites in the genome for different window sizes**. We plot –log_10_ P value of the likelihood ratio test for presence of effects of local ancestry proportions for different window sizes for each tested DNAm site in colorectum. Sites which are significant (FDR 1%) are shown in red.

**
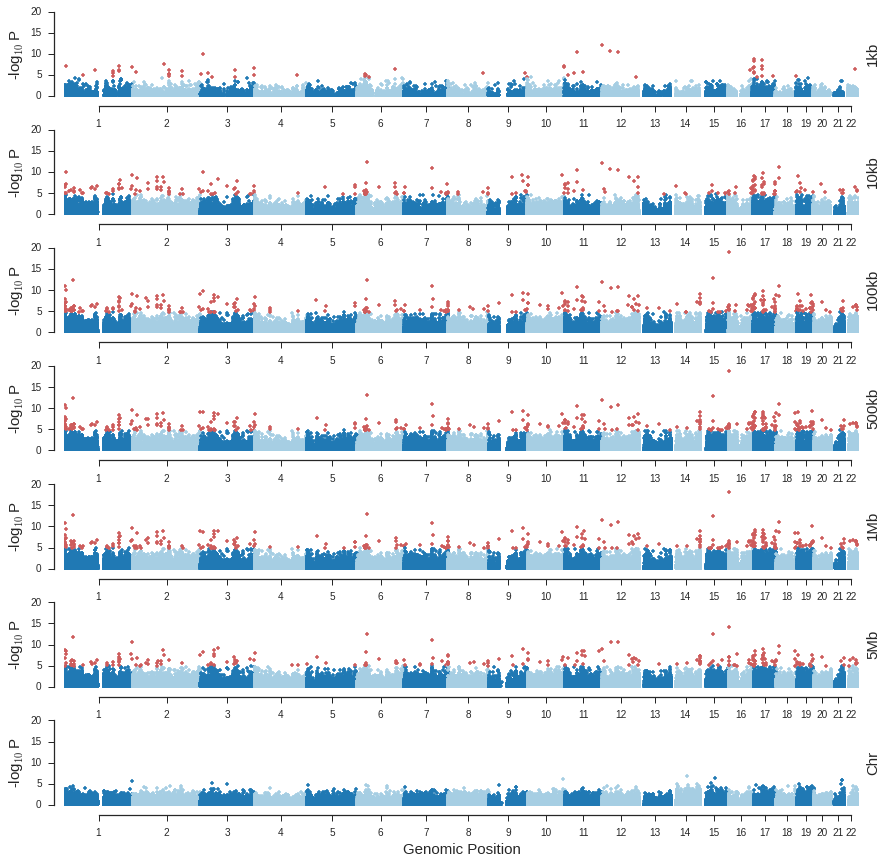
**

**Supplementary Figure 4.** **Distribution of SNPs within a 500kb window across DNAm sites.** Only sites which contain at least one SNP are included. We plot the histograms for sites which did and did not show a significant effect (FDR 1%) of local ancestral population in a 500kb window in colorectum.


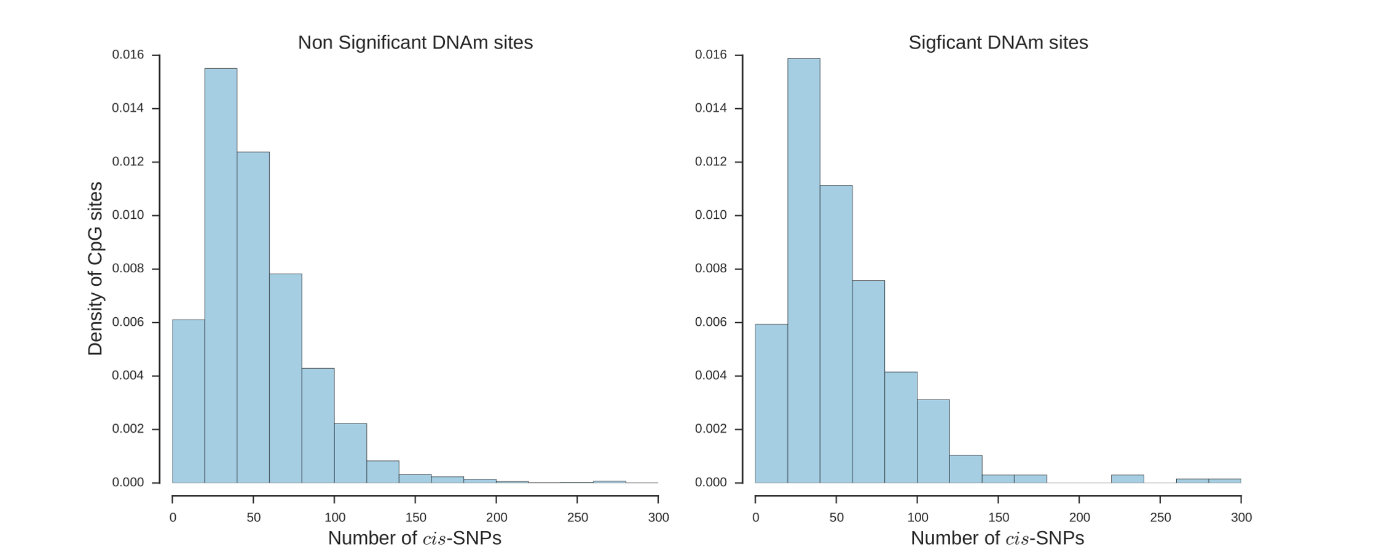


**Supplementary Figure 5. Effect of genome-wide ancestry on popDNAm site discovery**. We compare the numbers of sites identified as popDNAm in the colorectum depending on the inclusion of genome-wide ancestry (GWA) as a covariate. We plot the numbers of sites with significant effect (FDR 1%) for each window size considered for local ancestry estimation as the absolute number (**a**) and as the fraction of sites tested for a specific window size (**b**).

**
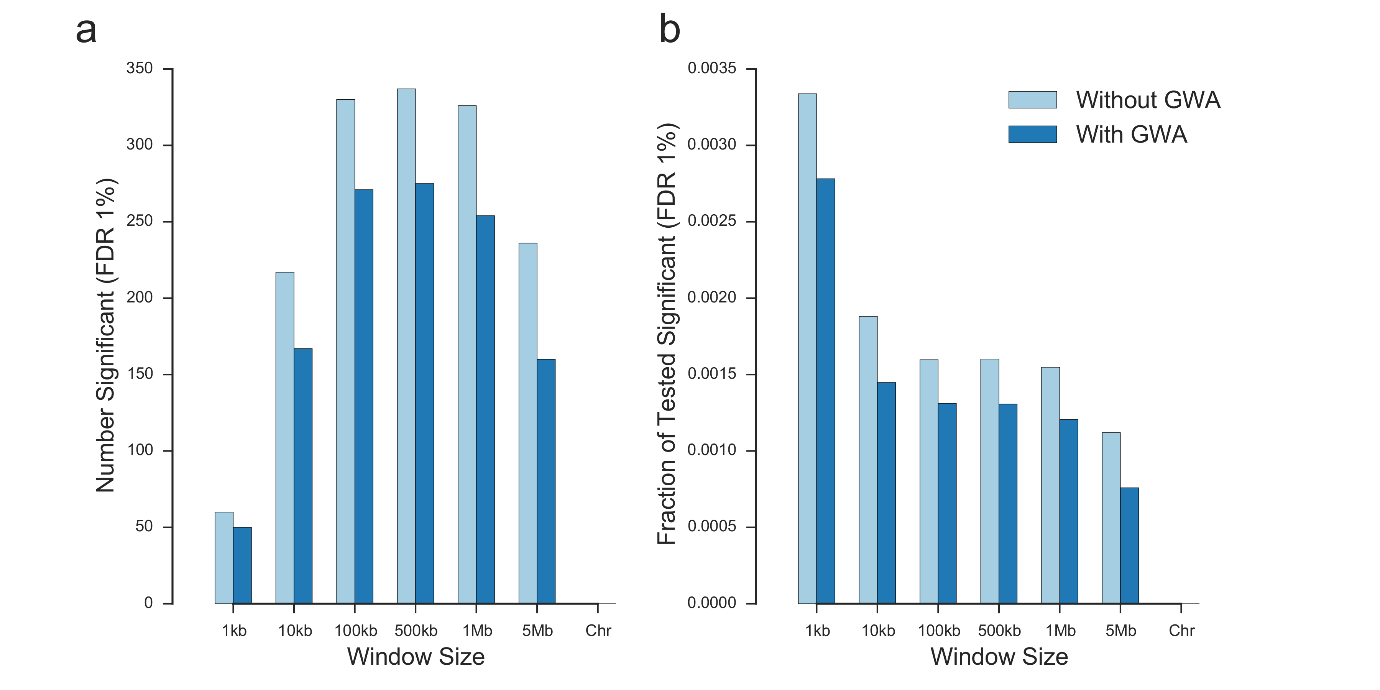
**

**Supplementary Figure 6:** Summary of popDNAm sites in colon for different significance thresholds. We plot the numbers of sites with significant effects of local ancestry for different window sizes used for local ancestry estimation for two different FDRs. Specifically, we show the absolute number of popDNAm sites (**a**) and the number of popDNAm sites as the fraction of sites tested for a specific window size (**b**).

**
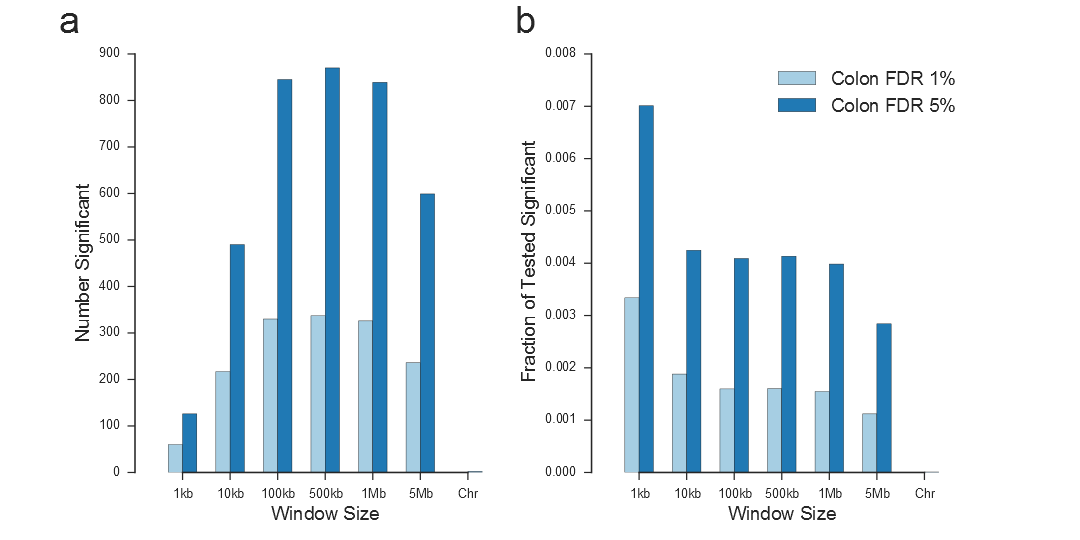
**

**Supplementary Figure 7. Enrichment of popDNAm sites towards the ends of chromosomes.** For DNAm sites located within certain distances from the ends of chromosomes we plot the fraction of sites which showed a significant (FDR 1%) effect of local ancestry based on a 500kb window in each of the two tissues considered. Error bars indicate the 95% CI intervals based on a binomial distribution.


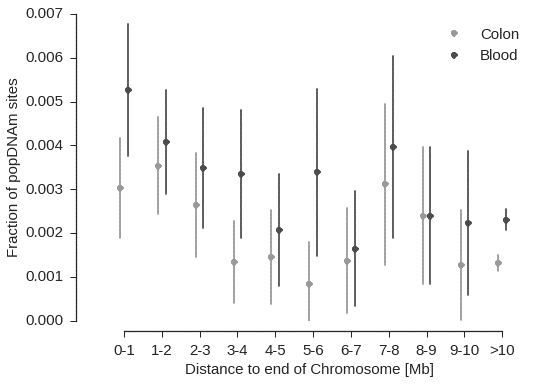


**Supplementary Figure 8.** **Effective number of individuals of pure local ancestry across DNAm sites**. Here pure local ancestry is defined as more than three-quarters local ancestry for one population base on a 500kb window.


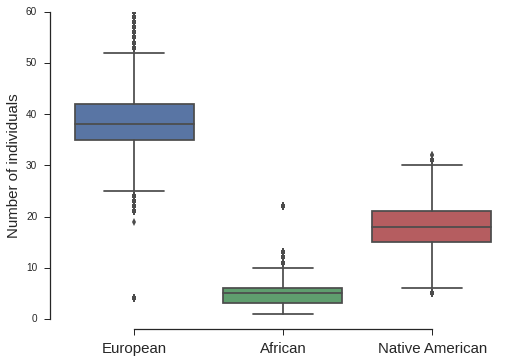


**Supplementary Figure 9. Effect of sample size on the ability to identify popDNAm sites.** Each sample size *N* corresponds to a comparison of *N* African to *N* European individuals. For each *N*, we plot the numbers of sites with a significant population effect (FDR 1%) across 10 subsamples of the data of Moen et al.. Horizontal lines indicate the number of sites we identified in our admixed population for either tissue using a window size of 500kb to estimate local ancestry.

**
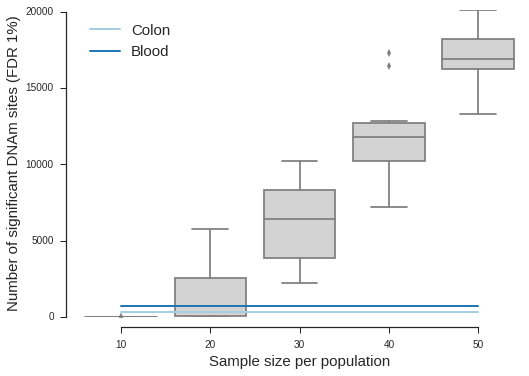
**

**Supplementary Table 2. Empirical P values for depletion and enrichment in relation to genes of popDNAm sites (500kb) in colorectum and blood.**

|  | **Colorectum** | | **Whole Blood** | |
| --- | --- | --- | --- | --- |
|  | **Depletion** | **Enrichment** | **Depletion** | **Enrichment** |
| **TSS1500** | 0.8011 | 0.1656 | 0.1764 | 0.7951 |
| **TSS200** | 0.0009 | 0.9983 | <0.0001 | 0.9999 |
| **5'UTR** | 0.0002 | 0.9997 | 0.0015 | 0.9982 |
| **1^st^ Exon** | 0.0001 | 0.9999 | <0.0001 | 0.9998 |
| **Body** | 0.4396 | 0.5139 | 0.5767 | 0.3892 |
| **3'UTR** | 0.0503 | 0.9025 | 0.0630 | 0.9003 |
| **Intergenic** | >0.9999 | <0.0001 | >0.9999 | <0.0001 |

**Supplementary Table 3.** **Empirical P values for depletion and enrichment in relation to CpG-Islands of popDNAm sites (500kb) in colorectum and blood.**

|  | **Colorectum** | | **Whole Blood** | |
| --- | --- | --- | --- | --- |
|  | **Depletion** | **Enrichment** | **Depletion** | **Enrichment** |
| **N. Shelf** | 0.4134 | 0.4772 | 0.5065 | 0.4107 |
| **N. Shore** | >0.9999 | <0.0001 | 0.7569 | 0.2091 |
| **Island** | <0.0001 | >0.9999 | <0.0001 | >0.9999 |
| **S. Shore** | 0.9925 | 0.0044 | 0.9981 | 0.0014 |
| **S. Shelf** | 0.3391 | 0.5485 | 0.4523 | 0.4598 |
| **Sea** | 0.4967 | 0.4555 | >0.9999 | <0.0001 |

**Supplementary Figure 10. Enrichment of popDNAm sites in whole blood for genetic context**. Specifically genes (**a**) and CpG islands (**b**). We plot the observed fraction of sites associated with a specific context amongst sites with a significant effect of local ancestral population and the expected such fraction under the assumption that such sites are randomly distributed ($H_{0}$). Error bars for $H_{0}$ indicate the empirically estimated 95% interval for this model. Considered sites showed a significant effect based on 500kb window in whole blood.

**
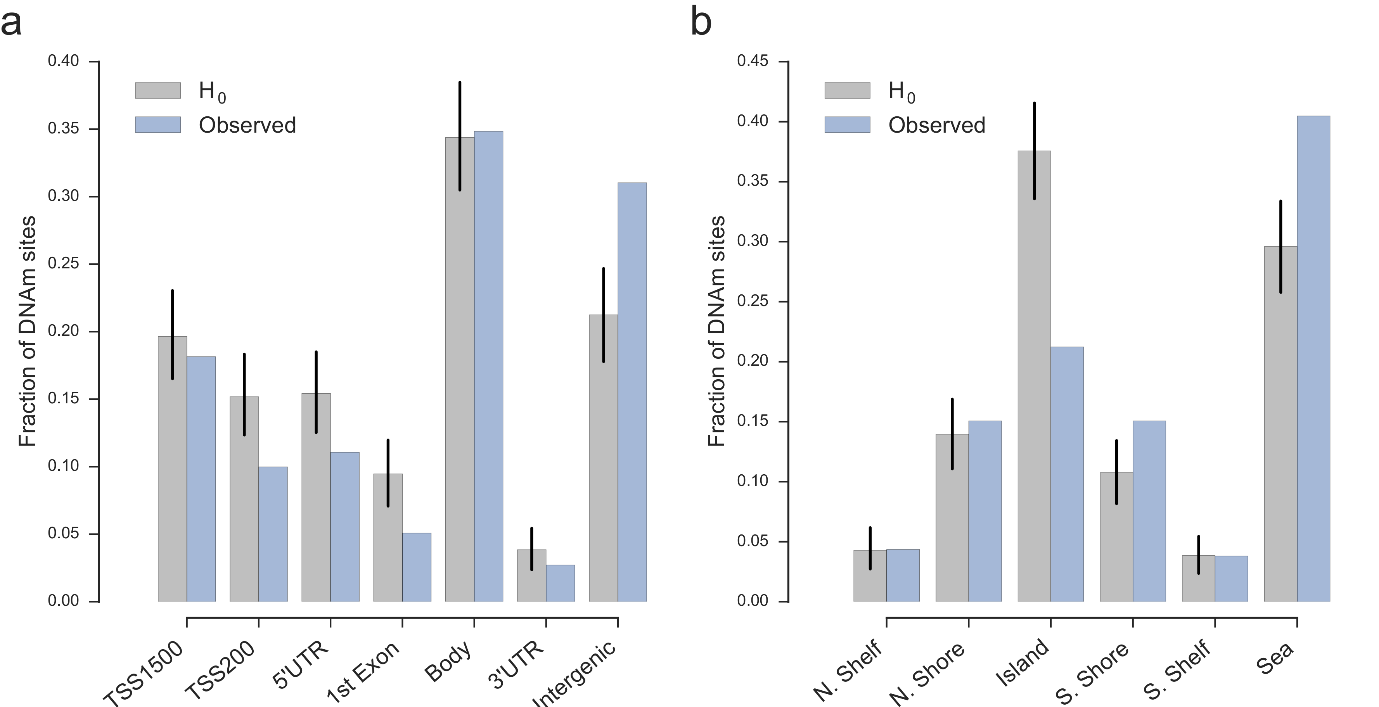
**

**Supplementary Figure 11. Enrichment of popDNAm sites further than 2Mb away from the ends of chromosomes in colon and whole blood for genetic context**. Specifically genes (**a**) and CpG islands (**b**) in colon and genes (c) and CpG Islands (d) in whole blood. We plot the observed fraction of sites associated with a specific context amongst sites with a significant effect of local ancestral population and the expected such fraction under the assumption that such sites are randomly distributed ($H_{0}$). Error bars for $H_{0}$ indicate the empirically estimated 95% interval for this model. Considered sites showed a significant effect based on 500kb window in whole blood.

**
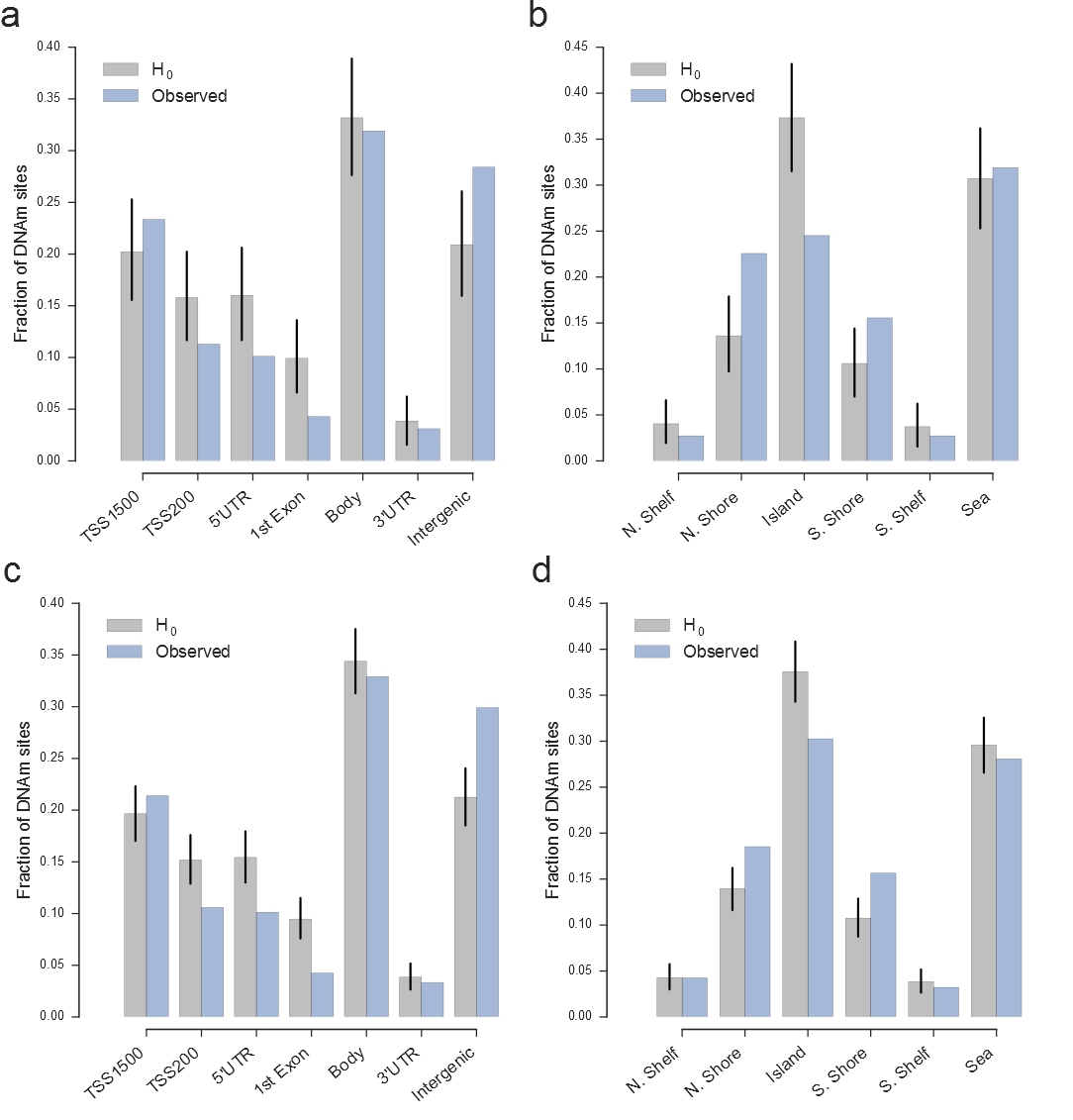
**

**Supplementary Figure 12. Enrichment of popDNAm sites at a less stringent significance threshold (FDR 5%) in colon and whole blood for genetic context**. Specifically genes (**a**) and CpG islands (**b**) in colon and genes (c) and CpG Islands (d) in whole blood. We plot the observed fraction of sites associated with a specific context amongst sites with a significant effect of local ancestral population and the expected such fraction under the assumption that such sites are randomly distributed ($H_{0}$). Error bars for $H_{0}$ indicate the empirically estimated 95% interval for this model. Considered sites showed a significant effect based on 500kb window in whole blood.

**
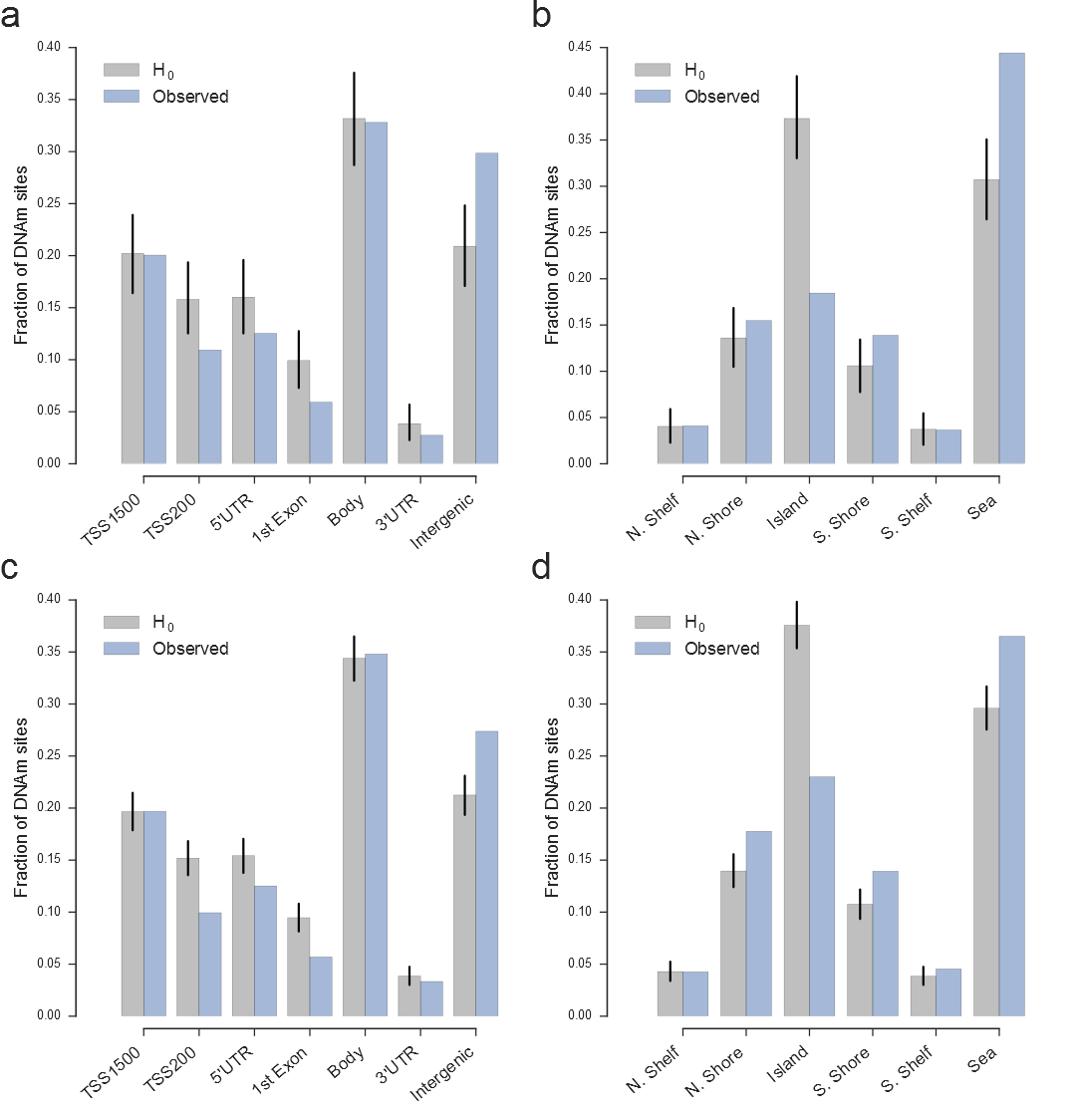
**
